# Uses of Fecal Microbiota Transplantation in Neurodegenerative Disease: A Scoping Review

**DOI:** 10.7759/cureus.62265

**Published:** 2024-06-12

**Authors:** Jenna Sanzone, Mason Life, Devan Reiss, Daniel May, Brianna Hartley, Patrick Spiddle, Joseph Al-Kirwi, Tigran Grigoryan, Joshua Costin

**Affiliations:** 1 Osteopathic Medicine, Nova Southeastern University Dr. Kiran C. Patel College of Osteopathic Medicine, Fort Lauderdale, USA; 2 Medical Education, Nova Southeastern University Dr. Kiran C. Patel College of Allopathic Medicine, Fort Lauderdale, USA

**Keywords:** parkinson's disease, treatment, multiple sclerosis (ms), dementia, neurodegenerative disesase, fecal microbiota transplantation (fmt)

## Abstract

Fecal microbiota transplantation (FMT) is the administration of fecal bacteria from a healthy donor into the intestinal tract of a recipient in order to directly change the recipient's gut microbial composition and confer a health benefit. The relationship between the gut microbiome and the central nervous system, termed the gut-brain axis, has been a frequent topic of gut microbiome studies. Commensal gut bacteria communicate with the central nervous system through various hormones, cytokines, and neural pathways. Therefore, influencing the gut microbiome via FMT may have the potential in treating symptoms of neurodegenerative conditions. This study aims to identify current uses of FMT in treating neurodegenerative diseases and highlight areas of future investigation. Following the Preferred Reporting Items for Systematic Reviews and Meta-Analyses (PRISMA) framework, a literature search was conducted of peer-reviewed sources on September 27, 2022, from Embase, MEDLINE, Web of Science, and Cochrane Central. Search terms were utilized that were related to the application of FMT and neurodegenerative disorders and limited those human studies, those that were published in English, and those that were published between 2017 and 2022. The initial search yielded 450 unique articles, and after the assessment of the title and abstract for inclusion and exclusion criteria, six articles were identified for full-text review. Studies that focused on either Parkinson's disease (PD) or multiple sclerosis (MS) demonstrated improvements in both motor symptoms and non-motor symptoms. FMT was also shown to provide significant relief of constipation and general gastrointestinal (GI) symptoms in all conditions studied. The studies related to MS showed the most mixed results with regard to symptomatic improvement. The data on the use of FMT as a treatment for neurodegenerative disorders is limited; however, studies have shown not only improvement in GI symptoms but also improvement in the cognitive symptoms of PD and dementia. The data on FMT as a treatment to improve the motor symptoms of PD is both more complete and more compelling than the data on the motor symptoms of MS. The studies that were reviewed showed no major adverse effects of FMT and generally promising results. There is a strong case to be made for larger, more well-controlled studies to be done on FMT and its potential use as a treatment not only for GI symptoms but for the motor and cognitive symptoms of neurodegenerative diseases.

## Introduction and background

The gut microbiome, consisting of approximately 10^14^ microorganisms, is a crucial component of the human gastrointestinal (GI) tract that primarily thrives in the less acidic, more oxygen-balanced environment of the colon [1,2]. These resident microorganisms play a pivotal role in human health. They are essential for synthesizing vitamins B1-B12, vitamin K, and vital amino acids that the body cannot produce independently [2,3]. Furthermore, the gut microbiome serves as the body's primary defense against pathogens, with certain bacteria generating bacteriocins that inhibit harmful bacterial proliferation. The microbiome as a whole also competes with exogenous bacteria for nutrients and adhesion sites and produces metabolic byproducts like short-chain fatty acids (SCFAs) [2,3]. These SCFAs provide energy to intestinal cells, bolstering the mucosal barrier against unwanted compound translocation into the bloodstream, and play a significant role in regulating local and systemic immunity. They enhance anti-inflammatory activity while reducing pro-inflammatory responses and autoimmune reactions, thereby modulating the body's immune responses to prevent detrimental effects [4].

Dysbiosis, on the other hand, is an imbalance in the microbiome composition, resulting in increased membrane permeability and overactive immune responses, which can allow disease to develop. The gut microbiome and dysbiosis play roles in the pathogenesis of cardiovascular disease, type II diabetes mellitus, and cancer, amongst others, though the mechanisms are not yet fully understood [1]. The various systems of the human body are all affected in different ways by fluctuations in the gut microbiota, and these gut-system relationships are the subject of recent research efforts.

Thus far, the relationship between the gut and the central nervous system (CNS), termed the gut-brain axis, has been a frequent topic of study. Commensal GI bacteria communicate with the CNS through the endocrine hormone system, immune system cytokines, and neural pathways, including the afferent vagus nerve, the enteric nervous system (ENS), and spinal nerves [5]. One notable discovery by Margolis et al. is that the GI system contains 90% of the body's total serotonin, a neurotransmitter found throughout the body [5]. Serotonin directly modulates GI functions of motility and secretion and allows crosstalk between the brain and the gut [6]. Spore-forming bacteria influence serotonin synthesis and secretion through their metabolic products, SCFAs [5]. These SCFAs stimulate the rate-limiting enzyme tryptophan hydroxylase (TPH) to synthesize serotonin, which is then secreted by endocrine cells within the GI tract, called enterochromaffin cells [5,6]. Serotonin can then stimulate additional gut microbes, like *Turicibacter sanguinis*, to take up serotonin and promote competitive colonization amongst the gut microbiota [5]. SCFAs also enter circulation, cross the blood-brain barrier, and then accumulate in the brain. As the SCFAs accumulate, they acidify the intracellular environment and may alter Ca^2+^ release, which in turn is thought to dysregulate neurotransmitter release and activate the immune system [7]. The exact mechanisms of communication between the gut microbiome and the CNS are not fully elucidated; however, a dysfunctional gut-brain axis has been postulated to play a role in the pathology of well-known neurodegenerative diseases, such as multiple sclerosis (MS), Parkinson's disease (PD), and dementia.

MS is a chronic inflammatory autoimmune demyelinating disease of the CNS [8]. There are four forms of MS seen clinically: relapsing-remitting MS, primary progressive MS, secondary progressive MS, and progressive-relapsing MS. Patient symptoms can include changes in vision, weakness, sensory loss or changes, dyscoordination, and changes of bladder/bowel function [8]. Both genetic and environmental factors have been proposed as contributing risk factors for MS. Recent studies on mice showed that alteration of metabolic activity induced by the gut microbiome and its associated lymphatic tissue affect the severity of CNS inflammatory demyelination [9]. Preliminary studies have also shown that the gut microbiome appears to be altered in those with relapsing types of MS [9].

PD primarily results from the death of neurons producing dopamine in the substantia nigra [10]. Genes linked to PD have led to the hypothesis that misfolding of proteins and dysfunction of the ubiquitin-proteasome pathway are involved in the pathogenesis of the disease, along with mitochondrial dysfunction and oxidative stress on dopaminergic neurons [10]. There is an increasing level of evidence showing that gut dysbiosis can influence the progression and onset of PD by increasing intestinal permeability, aggravating neuroinflammation, and aggregating abnormal levels of α-synuclein fibrils, consequently increasing oxidative stress and decreasing neurotransmitter production [11].

Dementia is a syndrome characterized by a cognitive decline significant enough to interfere with activities of daily living [12]. Numerous underlying etiologies can result in dementia. Neurodegenerative dementias, such as Alzheimer's disease, are more common in the elderly, whereas traumatic brain injuries and brain tumors are common causes in younger adults [12]. Research has shown that patients with mild cognitive impairment and Alzheimer's disease present with several metabolic and immune-inflammatory alterations, which are thought to affect the composition of the gut microbiome resulting in dysbiosis [13]. This gut dysbiosis results in abnormal signaling and protein formation with subsequent aberrant metabolic processes relevant to the development and progression of dementia [13].

The gut microbiome can be altered by many variables, including nonspecific host factors that affect the ability of commensal bacteria to proliferate along the GI lumen and mucosal lining, as well as specific host factors that directly regulate the capacity of bacteria in the gut to replicate [14]. The composition of one's microbiome can additionally be affected by age, mood/stress level, method of delivery at birth (vaginal versus cesarean section), diet, antibiotic usage, and a host of other factors [1,15,16]. The microbiota of the gut can also be directly modulated through the use of probiotics, prebiotics, and fecal microbiota transplantation (FMT) which can all be used to bring balance back to the gut microbiome. Probiotics are living organisms that can be ingested in specific dosages to help support gut health. These organisms can prevent the replication and adhesion of harmful bacteria, and certain species of bacteria within probiotics can even produce compounds capable of killing pathogenic bacteria [17,18]. Prebiotics are fermentation components, generally non-digestible carbohydrates, that are meant to incite the growth or activation of particular types of bacteria to benefit health [19].

FMT involves the administration of fecal bacteria from a healthy donor into the intestinal tract of a recipient to directly change the gut microbial composition and confer a health benefit [20]. Literature suggesting the use of FMT to treat food poisoning dates back to the fourth century. In modern medicine, Eiseman et al. first reported the use of FMT to treat pseudomembranous colitis in 1958. There has been an increasing use of FMT in patient care in recent years. Past research focused primarily on recurrent *Clostridioides difficile* infections (CDIs), with cure rates as high as 90% [21]. Antibiotics are commonly prescribed to treat pathogenic bacterial infections, such as CDIs. However, the use of these medications also results in the death of the beneficial bacteria that make up the gut microbiome, resulting in the dysbiosis and overgrowth of harmful bacteria (including CDI) that are not sensitive to the antibiotic [22]. FMT restores the balance of bacteria in the gut, thereby eliminating the opportunity for harmful bacteria to overgrow.

With recent advancements in healthcare, the United States is experiencing a demographic shift regarding the age of its citizens, with one in four people being 65 years of age or older by the year 2060 [23]. The aging of the US population over time is correlated with an increased incidence of neurodegenerative disorders, creating a need to explore the therapeutic potential of FMT in this area. Current research using FMT to directly alter the microbiome focuses on a wide range of disease processes, from inflammatory bowel disease to autism. The intention of this scoping review is to specifically identify current research on the uses of FMT to treat neurodegenerative diseases. As described above, the effects of the gut microbiome on human health and wellness are potential therapeutic targets for many areas of medicine. This review will additionally identify potential gaps in that research.

## Review

Methods

Eligibility Criteria

To be included in this review, articles had to be written in English, with research conducted anywhere in the world. The included papers were restricted to those involving human subjects receiving FMT as a treatment for neurodegenerative disorders. All routes of FMT administration, including nasogastric (NG) tube, nasojejunal (NJ) tube, esophagogastroduodenoscopy (EGD), coloscopy, and retention enema, were included. The search was limited to primary research studies (including randomized clinical trials and single-arm uncontrolled studies) that took place between 2017 and 2022 to ensure that the review provided an accurate picture of current research. All reviews were excluded, as well as papers that discussed topics associated with the use of FMT in neurology and psychiatry outside of neurodegenerative disorders.

Initially, the authors planned to limit their scoping review to peer-reviewed studies conducted within the United States, with the goal of better understanding the landscape of FMT in their eventual area of practice as future US physicians. However, because of the paucity of research on this topic within the United States, the research scope was expanded to include research in all countries.

Information Sources and Search Strategy

To define the research question, the Population, Concept, and Context (PCC) framework was utilized, and the study was carried out using standard Joanna Briggs Institute (JBI) guidelines for conducting scoping reviews [24]. The population was defined as males and females of any age; the concept was the use of FMT in neurodegenerative diseases; and the context was FMT use throughout the world from 2017 to 2022. The following electronic databases were used for data collection: MEDLINE (Ovid), Embase, Cochrane Central, and Web of Science. Ovid MEDLINE was chosen for its broad range of literature related to biomedicine and the allied health fields, as well as for its greater ability to perform more focused searches in comparison to PubMed. Embase is yet another extensive source of biomedical literature, capable of focused searches. Cochrane Central offers a unique ability to access only randomized and quasi-randomized controlled trials. Lastly, with the Web of Science database, a wide net could be cast over multiple disciplines and various journals. No sources of gray literature were used.

Using the PCC framework, a search string was created. The search string incorporated Boolean operators AND, OR, NEAR, and truncation/wildcard (symbol *), combined with keywords, Medical Subject Headings (MeSH), synonyms, and spelling variations of all relevant terms indicated in the PCC framework. Limitations were set to include only human studies (if permitted by the database) and literature published in 2017-2022. These limitations were set to focus on the most recent and clinically applicable literature. Initial data collection from Embase was conducted on September 27, 2022, and translation of those search terms to the remaining databases occurred on September 30, 2022. The search was not supplemented with scanning of reference lists of relevant reviews or hand-searching key journals. Table 1 indicates the exact search terms and the order they were applied during data collection from Embase. These terms and exact order were translated to the remaining three databases using the preferred search syntax for each. An abridged de-duplication methodology was applied to this scoping review using EndNote [25]. The Preferred Reporting Items for Systematic Reviews and Meta-Analyses (PRISMA) flow diagram was utilized to record identified, included, and excluded articles as the authors progressed through the different phases of the scoping review process [26].

**Table 1:** Embase search strategy

Embase search strategy	
1	‘fecal microbiota transplantation’/exp
2	(fecal OR faecal OR feces OR gut OR stool) NEAR/2 (transplant* OR transfusion* OR instillation* OR enema* OR infusion*)
3	‘alpers disease’/exp OR ‘alzheimer disease’/exp OR ‘chorea-acanthocytosis’/exp OR ‘chronic traumatic encephalopathy’/exp OR ‘corticobasal degeneration’/exp OR ‘diffuse neurofibrillary tangles with calcification’/exp OR ‘friedrich ataxia’/exp OR ‘frontotemporal dementia’/exp OR ‘hippocampal sclerosis’/exp OR ‘mcleod syndrome’/exp OR ‘neurodegeneration with brain iron accumulation’/exp OR ‘parkinson disease’/exp OR ‘perry sydrome’/exp OR ‘pick presenile dementia’/exp OR ‘retina degeneration’/exp OR ‘senile dementia’/exp OR ‘striatonigral degeneration’/exp OR ‘subacute combined degengeration’/exp OR ‘synucleinopathy’/exp OR ‘tauopathy’/exp
4	‘dementia’/exp OR ‘amyotrophic lateral sclerosis’/exp OR ‘multiple sclerosis’/exp OR ‘progressive supranuclear palsy’/exp
5	dementia:ab,ti,kw OR parkinson*:ab,ti,kw OR alzheimer*:ab,ti,kw OR ‘amyotrophic lateral sclerosis’:ab,ti,kw OR ‘multiple sclerosis’:ab,ti,kw OR huntington*:ab,ti,kw OR ‘progressive supranuclear palsy’:ab,ti,kw OR neurodegenerat*:ab,ti,kw
6	#1 OR #2
7	#3 OR #4 OR #5
8	#6 AND #7
9	#6 AND #7 AND [humans]/lim
10	#6 AND #7 AND [humans]/lim AND (2017-2022)/py

Prior to the title and abstract review, inter-rater reliability was assessed amongst the authors in order to assure all were clear regarding the inclusion and exclusion criteria. Ten of the 450 articles were randomly selected and reviewed, and the results were discussed. Any discrepancies were elaborated upon, with the members coming to a consensus.

A data charting form was created by authors using Microsoft Excel to guide which variables would be extracted. Authors 6 through 8 independently charted the data and then compared and updated results after thoroughly discussing any apparent discrepancies. The items selected for charting were those determined to be most relevant to the previously established criteria. Data was abstracted based on article characteristics (e.g., country of origin, year of publication), contextual factors (e.g., population characteristics, type of study, route of FMT application, success rate of FMT), and relevance to the concept (use of FMT in neurodegenerative diseases).

The six articles selected from the full-text reviews were evaluated for quality using the JBI critical appraisal tools. Articles scoring above 70% were considered high quality, those scoring between 50% and 70% were considered medium quality, and all articles scoring below 50% were considered low quality.

Results

**Figure 1:**
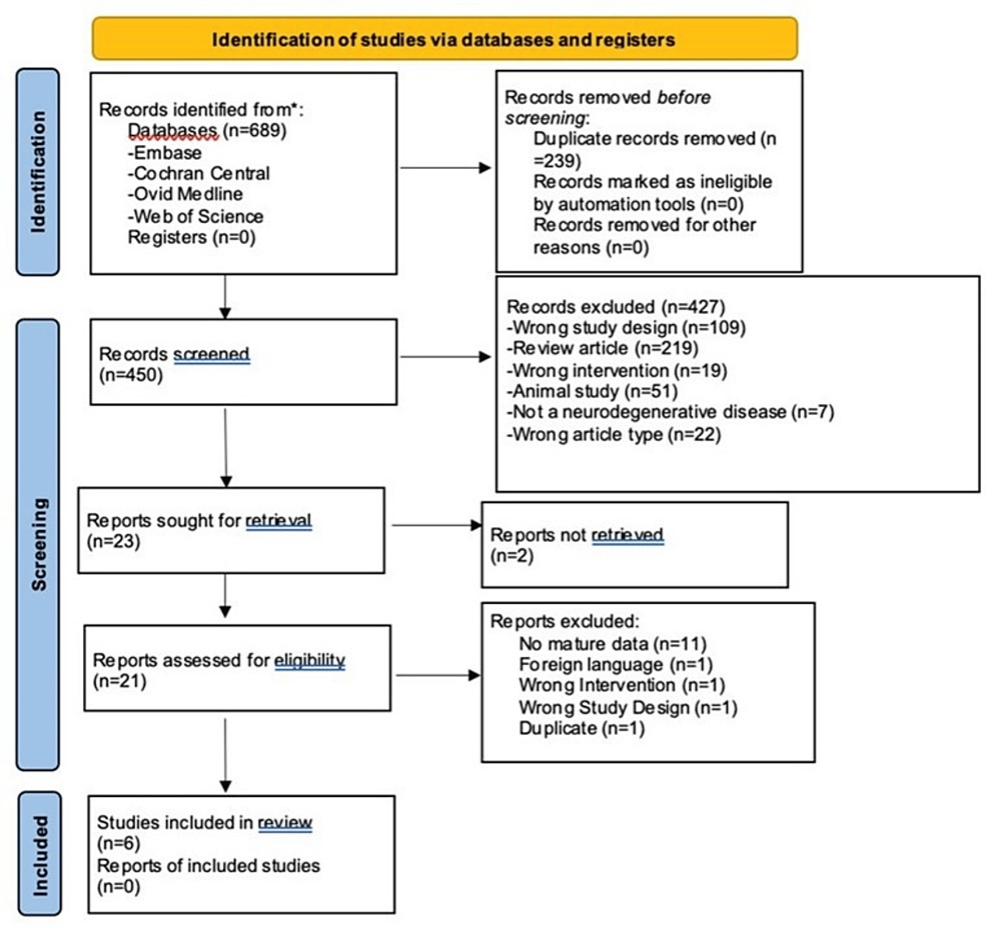
PRISMA flow diagram PRISMA: Preferred Reporting Items for Systematic Reviews and Meta-Analyses

A total of six articles, each below 50% quality, were identified that fit the inclusion criteria (Figure 1). Every study included in this review showed improvement after FMT treatment in at least one of the symptoms that the study targeted (Table 2). The studies were conducted in China (n=2), South Korea (n=1), Israel (n=1), Canada (n=1), and the United States/United Kingdom/Bahamas (n=1). FMT was used to target PD in three studies, MS in two studies, and dementia in one study, with the most significant symptomatic improvements seen in Parkinson's and dementia studies.

**Table 2:** Data extraction chart Data extracted from the included studies. FMT: fecal microbiota transplantation; MS: multiple sclerosis; SCFA: short-chain fatty acid; GI: gastrointestinal; DNA: deoxyribonucleic acid; PCR: polymerase chain reaction: PD: Parkinson's disease; BDNF: brain-derived neurotrophic factor; MSWS-12: 12-Item MS Walking Scale; PSQI: Pittsburgh Sleep Quality Index; HAMD: Hamilton Depression Rating Scale; HAMA: Hamilton Anxiety Rating Scale; PDQ-39: Parkinson's Disease Questionnaire; UPDRS-III: Unified Parkinson's Disease Rating Scale; NMSQ: Non-Motor Symptoms Questionnaire; H-Y: Hoehn and Yahr Scale; NMSS: Non-Motor Symptoms Scale; PAC-QOL: Patient Assessment of Constipation Quality of Life; BSS: Bristol Stool Scale; GCS: Glasgow Coma Scale; MMSE: Mini-Mental State Examination; CDR-SB: Clinical Dementia Rating Sum of Boxes; LHBT: lactulose hydrogen breath test; SIBO: small intestine bacterial overgrowth

Title	Study design	Participants (numbers and age ranges)	Location	Study aim	FMT method of administration	Neurodegenerative disease	Disease stage	Pre-treatment microbial taxa predominance	Post-treatment microbial taxa predominance	Time between FMT and evaluation	Measures used	Findings	Recommendations	Limitations	Notes
Single-arm, non-randomized, time series, single-subject study of fecal microbiota transplantation in multiple sclerosis (Engen et al., 2020 [27])	Single-arm, non-randomized, time series, single-subject	A 48-year-old Caucasian male	Chicago, IL, USA	Assess whether FMT interventions can alter the MS microbiome, decrease inflammatory biomarkers, and improve MS symptoms in a subject with relapsing-remitting MS and severe gait problem	Rectal catheter	MS	Active relapsing-remitting MS for two years	*Faecalibacterium prausnitzii,* *Collinsella aerofaciens,* *Eubacterium rectale*	*Faecalibacterium prausnitzii* significantly increased microbial parameters (*Firmicutes*-to-*Bacteroidetes* and *Prevotellaceae*-to-*Bacteroidaceae* ratios) and significantly increased putative butyrate-producing species *Collinsella aerofaciens* and *Eubacterium rectale* (but not significant)	Three weeks and every 13 weeks afterwards (a total of 12 months of observation)	SCFA metabolite concentrations (acetate, propionate, butyrate, total SCFA, and total butyrate-to-total SCFA ratio), serum BDNF; IL-6, IL-8, IL-17, TNF-a, gait questionnaire, objective gait metrics (gait, side gaze gait, alternating gaze gait, stride time, stride distance, cadence, step width, average pelvis forward velocity, and pelvis smoothness), MSWS-12, PROMIS® GI symptom scale, automated self-administered 24-hour, 24-hour recall Food Timing Screener, Food Timing Questionnaire	Lasting impact of FMT on the microbiome and clinical features of MS. FMT increased the numbers of deficient bacteria in the patient over time. Did not affect the level of serum inflammatory markers. Objective and subjective measures of gait and GI disturbance improved as well	FMT might be an emerging treatment in relapsing-remitting MS; need future randomized controlled trials	Single-subject, open-labeled design. No control over the FMT procedure administered by the Taymount Clinic	Controlled for dietary impact by analyzing spouse's fecal sample and having the participant monitor his dietary intake. Predominantly plant-based diet
Fecal microbiota transplantation is safe and tolerable in patients with multiple sclerosis: a pilot randomized controlled trial (Al et al., 2022 [28])	Randomized controlled trial	Nine treatments, four treated with early intervention and five with late intervention (three males and six females), with a mean age of 44±8.2 years. 10 healthy controls	London, ON, Canada	Investigate the safety and tolerability of FMT in MS patients and whether it can improve abnormal intestinal permeability	Rectal enema	MS	Relapsing-remitting MS	Donors had higher *Prevotella* and *Paraprevotella.* MS patients showed higher *Bacteroides*, *Blautia faecis*, and *Bacteroides uniformis* as well as higher pro-inflammatory cytokines	Statistically significant alterations in flora that were seen were donor-specific	1× per month for up to 12 months	Blood work, urinalysis, vitals, cytokine analysis, MRI (baseline, six months, and 12 months), Expanded Disability Status Scale, intestinal permeability (baseline, six months, and 12 months), DNA amplification via PCR, fecal sample collection, Shannon's index of alpha diversity	No significant change in the levels of any of the cytokines measured post-FMT. No significant change in fecal microbiota diversity. Previously elevated small intestinal permeability was normalized following FMT. Associated with significant alterations in the gut microbiota. Increase in the anti-inflammatory electron carrier ubiquinone	FMT was a safe and tolerable intervention in this group of MS patients and has the potential to normalize intestinal permeability and produce durable, beneficial alterations to the gut microbiota	Low sample size that is more representative. No bowel prep or antibiotics prior to initial FMT procedure. Did not control for diet	Premature study termination. Analyzed microbiota composition in the participants six months prior to and after FMT
Fecal microbiota transplantation therapy for Parkinson's disease: a preliminary study (Xue et al., 2020 [29])	Case series	15 total PD patients. Colonic FMT group: 10 received FMT via colonoscopy (seven males and three females). Nasointestinal FMT group: five received FMT via nasal-jejunal tube (four males and one female). No control. Ages 49-72	China	Assess the efficacy and safety of FMT on PD	Colonoscopy, nasal-jejunal tube	PD	Disease duration ranged from two years to 13 years (median=4 years). H-Y Scale: ranging from 1.5 to 4 (median=3)	N/A	N/A	One and three months of follow-ups. All 15 participants completed the one-month follow-up. 12 participants completed the three-month follow-up	PSQI, HAMD, HAMA, PDQ-39, UPDRS-III, NMSQ	PSQI, HAMD, HAMA, PDQ-39, and UPDRS-III significantly decreased after FMT treatment. Colonic FMT group showed significant improvement and longer maintenance of efficacy compared with nasointestinal FMT	Placebo randomized controlled trial to further study the efficacy and safety of FMT	No control group. Preliminary study with few cases. The follow-up period is short. No analysis of microbiome profile or inflammatory markers	Discontinuation of drug regimen throughout the study
Evaluation of fecal microbiota transplantation in Parkinson's disease patients with constipation (Kuai et al., 2021 [30])	Prospective single study	11 treatment. No control. Ages 40-84 with a mean age of 62.45 (four females and seven males)	Suzhou, Jiangsu, China	Evaluate the effectiveness and safety of FMT for PD patients with GI dysfunction	Nasoduodenal tube	PD	Varying stages of PD with confirmed symptoms of constipation using the Wexner Constipation Score. Disease duration ranging from one year to 12 years (median=7.18±3.25 years)	Phylum *Bacteroidetes*, genus *Bacteroides*, order *Bacteroidales*, class *Bacteroidia*, and family *Bacteroidaceae* were the dominant bacteria	*Coriobacteriaceae*, *Erysipelotrichaceae,* and *Lachnospiraceae*, belonging to the phylum *Actinobacteria* and *Firmicutes,* respectively	Six weeks and 12 weeks of follow-ups. Fecal sample collection before and four, eight, and 12 weeks after FMT	H-Y Grade, UPDRS-II Score, NMSS, PAC-QOL score, Wexner Constipation Score, body mass index, homocysteine, albumin, and uric acid	H-Y Grade, UPDRS-II Score, and NMSS of PD patients decreased significantly after FMT. LHBT indicated that SIBO returned to normal. PAC-QOL score and Wexner Constipation Score in after‑FMT patients decreased significantly	Complete a future study with a larger sample size. FMT can be used for Parkinson's treatment but safety/effectiveness needs further evaluation	A small number of participants varying stages of disease	The drug regimen continued throughout the study
Fecal microbiota transplant as a potential treatment for Parkinson's disease – a case series (Segal et al., 2021 [31])	Case series	Six PD patients aged 47-73 (three males and three females)	Israel	Determine whether FMT is safe and possibly efficacious in treating constipation and motor and non-motor symptoms in PD patients	Colonoscopy	PD	Disease duration ranging from 1.5 years to 15 years. H-Y Scale: five of six participants with a score of 2 and one of six with a score of 1	N/A	N/A	Two, four, eight, 12, 16, 20, and 24 weeks	UPDRS-III, H-Y, NMSS for PD, Wexner Score, BSS	FMT via colonoscopy was safe and resulted in the improvement of PD motor and non-motor symptoms	Further research needed to assess longer-term maintenance of efficacy and safety, including in large-scale randomized controlled trials	Small sample size. No control or blinding. Short follow-up period. No analysis of microbiome profile or inflammatory markers	
Fecal microbiota transplantation can improve cognition in patients with cognitive decline and *Clostridioides difficile* infection (Park et al., 2022 [32])	Randomized controlled trial	10 treatment. Ages 63-90 (eight females and two males). 10 control. Ages 62-91 (eight females and two males)	Republic of Korea	Establish that FMT leads to significant improvements in cognitive performance and discuss the relationship between cognition and the gut microbiome	Colonoscopy	Dementia, especially Alzheimer's disease	Mild to moderate dementia	DQ805799_s *Staphylococcus*, *Staphylococcaceae*, *Staphylococcus aureus* group	*Clostridiales,* *Clostridia,* *Erysipelotrichia,* *Erysipelotrichales,* *Erysipelotrichaceae*	One month	GCS, MMSE, CDR-SB	Significant improvement on MMSE performance and significant reduction in CDR-SB scores for all 10 patients in the treatment group. Control group did not show statistically significant cognitive function after (curative for 9/10) antibiotic treatment for *C. difficile.* No significant change in GCS for pre-/post-transplant in the treatment or control groups. FMT may effectively delay cognitive decline in patients with dementia	Complete long-term study	Short-term study. No brain imaging. Small sample size	FMT was curative for *C. difficile* infection in nine patients after one transplant and for the remaining patient after two transplants

FMT Administration

The routes of FMT administration varied, with two of the six studies using colonoscopy, one using a rectal catheter, one using a rectal enema, one not specifying the method, and one comparing the use of both colonoscopy and nasointestinal tube. The studies also varied in sample preparation, bowel portion targeted, and post-administration instructions for patients. In most studies, the transplant was completed in one day, while in two studies, it was a multi-step process (one FMT per month for six months and then the other with a second FMT between 26 and 39 weeks after the initial treatment) [27,28]. It is difficult to assess which (if any) of these differences was significant across studies, although it is notable that in the study that contrasted colonoscopy and nasointestinal administration, only the colonic FMT group showed significant symptomatic improvement [29].

Microbial Taxa Predominance

While not every study reported on microbial taxa both pre- and post-FMT, the four that did reported a change in species predominance. It is notable that in two of those studies, the *Bacteroides *species were predominant pre-transplant and then subsequently decreased post-transplant [28,30]. This demonstrates that FMT was successful in at least changing the microbial environment of the GI tract in these studies.

PD

PD is the neurodegenerative disease that shows the most promising symptomatic results based on the available data. In each of the three studies analyzed, patients showed significant improvement in GI motility, motor symptoms, and non-motor symptoms (which include cognitive, emotional, psychological, and sleep). A notable exception is the nasointestinal FMT treatment group in Xue et al.'s study, which did not show statistically significant improvement by most measures and included a high rate of dropout due to patient dissatisfaction with their treatment protocol [29].

The PD studies are additionally illustrative in that they were longitudinal designs. In the earliest assessment performed at two weeks post-treatment, improvement was apparent in several categories comprising constipation and non-motor symptoms [29-31]. All three studies showed continued improvement in some or all symptom categories between early and final assessments post-baseline [29-31].

Dementia

While only one study was included on dementia, it demonstrated significant potential benefits of using FMT as a treatment for dementia. In this study, patients with dementia with concomitant CDI were compared using CDI treatment with FMT as the treatment arm and CDI treatment with antibiotics as the control arm. While both treatments proved effective for CDI, all of the 10 patients in the treatment group also showed markedly significant improvement in their cognitive symptoms. No statistically significant improvement in cognitive symptoms was seen in the control group [32].

MS

Of all the studies assessed in this review, the two related to the FMT treatment of MS showed the most mixed results with regard to symptomatic improvement. One study's results are limited by early termination by the IRB due to the unexpected death of the principal investigator, and the other study was a single-subject design more focused on biochemical markers and biochemical diversity than symptomatic improvement. Neither study was controlled [27,28]. In the larger study, most symptomatic measures showed no significant improvement across multiple assessments during the six months of the study, although both patients with abnormal intestinal permeability at baseline saw this measure normalize by the study's conclusion. In the single-patient study, the patient's gait improved significantly, but it was already significantly improved (and improving) from assessments one and two years before the study. Therefore, it is difficult to ascertain whether the improvement in gait was related to treatment.

Discussion

No studies that met the inclusion criteria showed a worsening of symptoms for any of varying degrees of improvement across the three different neurodegenerative conditions examined (PD, MS, and dementia). Most included research actually showed a marked improvement in condition-specific symptoms and biomarkers after treatment with FMT. Studies that focused on PD demonstrated improvements in both motor symptoms and non-motor symptoms, whereas studies highlighting MS demonstrated a positive impact on gut permeability and overall gut microbiome health. FMT was also shown to provide significant relief of constipation and general GI symptoms in all conditions studied.

It was interesting to note that although several different methods of FMT administration were used across studies, this did not appear to significantly affect its efficacy. Since only one study directly compared treatment efficacy between two routes of FMT administration, there is no enough data for an analysis of FMT administration methods. However, because this study found a significant therapeutic effect with colonoscopy-administered FMT and no significant symptomatic improvement with NJ tube-administered FMT, more research should be performed on the impact of FMT methods in their treatment of neurodegenerative diseases. Those conducting further investigations should evaluate whether this effect is real, if it is consistent across different disease conditions, and what the ideal route of administration would be.

Frequency of administration is often thought to contribute to treatment outcomes. There was significant variation in the definition of what a single FMT administration consists of, as well as the time frame of administration (if multiple administrations were dictated by the study). Regardless, given that all included studies reported either successful or neutral outcomes, administration of more than one fecal transplant did not seem to significantly impact results.

Though the hypothesized trends in the data were not fully illustrated in this particular data set, the fact that each study had its own unique method of measuring outcomes, even between studies investigating the same neurodegenerative disease, made it difficult to appreciate any subtle differences in the degree of success of the treatments. A more standardized approach to measuring functional outcomes of a particular neurodegenerative disease, perhaps, would elucidate any differences based on the method or frequency of FMT administration.

Limitations in the Included Studies

One of the glaring deficiencies in the research regarding FMT use in treating neurodegenerative conditions is not only the limited number of studies but also the limited number of countries worldwide taking part in research of this nature and, notably, in the United States. Of the six studies examined in this review, only two were randomized controlled studies, and both of those had limited numbers of patients. There were no double-blind studies included, possibly due to the difficulty of the procedural nature of administering FMT. There was also variable control of the participants' diets across the studies, which is potentially a flaw in the design of multiple included papers since there is evidence that a patient's diet does directly affect the quality and diversity of their microbiome.

Limitations of the Review Process

In conducting this scoping review, there were a few limitations in the collection and synthesis of reliable data. The review was limited to just four databases, and if the search had been expanded to more databases, it may have generated more articles to analyze. Another limitation is the relatively few number of articles pertaining to FMT and its uses in neurodegenerative conditions. Initially, the inclusion criteria were restricted to studies conducted in the United States. However, given the lack of published research available in the United States, the criteria were expanded to include international studies.

Nonetheless, even with the expanded inclusion criteria, the review was limited to low-level evidence, with only two randomized controlled trials as mentioned above. In addition to the study design, all included studies used small sample sizes, with 20 total participants at the most. Given the absence of high-quality studies, it was determined not to exclude any studies based on quality, so as to have an accurate assessment of the relatively small body of research done on this important topic.

Furthermore, this scoping review's inclusion criteria limited research to only human studies. It is worth noting that there were many published animal studies found in the initial screen that were subsequently excluded. This implies that most of the current research has not yet progressed to human trials and remains in animal models.

Recommendations

Importantly, the current body of research in FMT as applied to neurodegenerative disease must grow and should focus on research designs with high levels of evidence such as double-blind randomized controlled trials. Human studies are scarce, and it is therefore difficult to make assumptions about the treatment's efficacy. A review published by Vendrik et al. in March 2020 looked at FMT as a treatment of neurological disorders in general (as opposed to neurodegenerative) and emphasized not just the lack of well-controlled, human studies but the lack of focus on safety [33]. As of November 30, 2022, the FDA approved the use of FMT in the treatment of patients with recurrent *C. difficile* infections [34]. With this being the first FDA-approved fecal microbiota product, data regarding the safety of this rectally administered FMT should dramatically increase. This product will potentially pave the way for further research in the United States regarding FMT and its future applications. Moreover, there is strong evidence that the food in one's diet modifies the gut microbiome and only one of the six identified studies attempted to control this confounding variable. The authors recommend that future studies control for diet.

## Conclusions

While the data on the use of FMT as a treatment for neurodegenerative disorders are limited, FMT represents an interesting and novel approach to a difficult problem. Given the significant overlap of nerve tracts, neurotransmitters, and cytokines in the gut-brain axis, it is unsurprising that FMT represents a fertile field of research for neurodegenerative diseases. And indeed, the studies that have been done show improvement not only in GI symptoms but also in cognitive symptoms of PD and dementia. Given the paucity of high-quality data on the use of FMT in neurodegenerative diseases, it would be premature to laud its use as a miracle treatment. Yet, the studies that were reviewed showed no major adverse effects of FMT treatment and generally promising results. There is a strong case to be made for larger, more well-controlled studies to be done on FMT and its use as a potential treatment not only for GI symptoms but for the motor and cognitive symptoms of neurodegenerative diseases.
